# Impact of *CYP2D6* genotype on fluoxetine exposure and treatment switch in adults and children/adolescents

**DOI:** 10.1007/s00228-025-03893-9

**Published:** 2025-08-08

**Authors:** Karoline Linnea Nordby, Pari Faraj, Espen Molden, Kristine Hole

**Affiliations:** 1 Center for Psychopharmacology, Diakonhjemmet Hospital, Oslo, Norway; 2 Department of Pharmaceutical Biosciences, School of Pharmacy, University of Oslo, Oslo, Norway; 3 Department of Life Sciences and Health, Oslo Metropolitan University, Oslo, Norway

**Keywords:** CYP2D6, Genotype, Fluoxetine, Pharmacogenetics, Therapeutic drug monitoring

## Abstract

**Purpose:**

The relevance of CYP2D6 activity on serum levels and clinical response of fluoxetine remains unclear. The present study aim was to evaluate the impact of *CYP2D6* genotype on i) fluoxetine and norfluoxetine exposure, and ii) treatment switch from fluoxetine to alternative antidepressants in adults and children/adolescents.

**Methods:**

Patients were included retrospectively from a therapeutic drug monitoring service. Patients were subgrouped by age, i.e. < 18 yrs (young) and ≥ 18 yrs (adult), and divided into CYP2D6 genotype-predicted phenotype subgroups of poor metabolizers (PMs), intermediate metabolizers (IMs), normal metabolizers (NMs) and ultrarapid metabolizers (UMs).

**Results:**

Among 1027 adult patients, the metabolic ratio was lowest in PMs and highest in UMs (*p* ≤ 0.04), but there was no difference in active moiety (fluoxetine + norfluoxetine) across CYP2D6 phenotype groups (*p* ≥ 0.1). Similar trends were observed in 196 young patients, both for metabolic ratio and active moiety. In adult patients, switching from fluoxetine to an alternative antidepressant had odds ratio of 2.9 in UMs (*p* = 0.004) and 2.3 in PMs (*p* = 0.007) compared with NMs. The number of switches per genotype group was too low for meaningful comparisons in young patients.

**Conclusion:**

The fluoxetine and norfluoxetine active moiety was unaffected by *CYP2D6* genotype in adults and children/adolescents. Still, adult CYP2D6 PMs and UMs switched antidepressant treatment two to three times more often than NMs, indicating that relative levels of fluoxetine and norfluoxetine may affect treatment outcome rather than active moiety in relation to CYP2D6 phenotype.

**Supplementary Information:**

The online version contains supplementary material available at 10.1007/s00228-025-03893-9.

## Introduction

Depressive and anxiety disorders are among the most disabling mental health disorders in the world [1]. Treatment of depression and anxiety comprises both pharmacological and non-pharmacological therapies. Selective serotonin reuptake inhibitors (SSRIs), introduced in the 1980 s, are the most used medications for treatment of these conditions [2]. Fluoxetine was the first SSRI to be approved by the FDA, and is used for the treatment of depression, obsessive–compulsive disorder, panic disorder, and bulimia nervosa [3]. Additionally, fluoxetine is the most frequently used SSRIs in children and adolescents, primarily due to its long history of use and extensive documentation on efficacy and tolerability [4]. However, it is unclear whether pharmacokinetic profiles of fluoxetine are similar in young and adult patients.

There is considerable variability in the efficacy of SSRIs, and 30–50% of patients do not respond to SSRI treatment [5]. Interindividual pharmacokinetic variability is one of the factors of importance for treatment response. Fluoxetine is demethylated to the pharmacologically active metabolite norfluoxetine, which has similar pharmacological activity as the parent compound [6]. Both in vitro*-* [7–10] and in vivo studies [11–15] indicate that cytochrome P450 (CYP) 2D6 is the main enzyme mediating the formation of norfluoxetine, with minor contributions from CYP2C9, CYP2C19 and CYP3A5 [8, 9]. The N-demethylation of fluoxetine is stereoselective. In vitro studies report that CYP2D6 is primarily responsible for the formation of S-norfluoxetine, while R-norfluoxetine is formed by both CYP2D6 and CYP2C9 [9, 11, 16]. Notably, S-norfluoxetine is approximately 20 times more potent as SSRI than R-norfluoxetine [15, 17].

CYP2D6 exhibits extensive pharmacogenetic variation, leading to variability in the serum concentration of several antidepressant drugs [18, 19]. However, for drugs with pharmacologically active metabolites, variation in enzyme activity does not necessarily lead to variation in active moiety, such as for the antipsychotic drug risperidone, where CYP2D6 mediates formation of the equipotent metabolite 9-hydroxyrisperidone. Despite limited effect on the overall pharmacologically active moiety, *CYP2D6* genotype has still been shown to significantly affect clinical outcome of risperidone in terms of increased treatment switch in CYP2D6 poor metabolizers (PMs) and ultrarapid metabolizers (UMs) [20]. This may reflect that the parent compound and metabolite exhibit different pharmacokinetic profiles, e.g. penetration across the blood–brain barrier, and also that ‘equipotency’ is a relative term [21].

For fluoxetine, several studies have demonstrated a significant correlation between the metabolic ratio and CYP2D6 phenotype [15, 22, 23], and higher concentrations of the parent compound have been reported in CYP2D6 PMs compared with normal metabolizers (NMs) [13, 14]. However, there is limited data assessing the active moiety of fluoxetine and norfluoxetine across CYP2D6 phenotype groups, although some studies report no change in active moiety both in adults [15, 22, 24] and in young patients [23]. Only a couple of studies have examined the relationship between *CYP2D6* genotype/phenotype and treatment response or side effects, yielding conflicting findings [25, 26]. To some extent, this may reflect an autoinhibitory effect, as both fluoxetine and norfluoxetine are competitive and reversible inhibitors of CYP2D6 [7]. This may cause phenoconversion of CYP2D6 metabolism in genotype-predicted intermediate metabolizers (IMs), NMs and UMs. While the elimination half-life of fluoxetine is usually within one to four days, it ranges from seven to 15 days for norfluoxetine [6].

Due to inconsistent findings in a limited number of small-scaled studies, international pharmacogenetic guidelines do not recommend individualized dosing based on *CYP2D6* genotype [18, 19]. To clarify the impact of genetic variability on CYP2D6-mediated metabolism of fluoxetine, both on relevant pharmacokinetic parameters and treatment outcomes, well-powered studies are required.

The aim of the present study was to evaluate the impact of *CYP2D6* genotype on i) individual variability in serum concentration of fluoxetine and its active metabolite norfluoxetine, and ii) treatment switch from fluoxetine to other antidepressants in adult patients and children/adolescents in a real-world patient population.

## Methods

### Patient inclusion

Patients were included retrospectively from a database including therapeutic drug monitoring (TDM) and pharmacogenetics analyses at Center for Psychopharmacology, Diakonhjemmet Hospital (Oslo, Norway). Fluoxetine/norfluoxetine serum concentrations with patient-matched *CYP2D6* genotypes were collected from the period 2010–2023. Relevant additional information was extracted from the TDM requisition forms, i.e., patient age, sex, fluoxetine daily dose, and the time interval between last dose intake and blood sampling. Information regarding patient ethnicity and body weight was not available from the requisition forms. We had no access to patient medical records and were therefore unable to retrieve clinical data.

Patient inclusion criteria were a) access to both fluoxetine serum concentration measurement and *CYP2D6* genotype for a patient; b) access to prescribed daily dose of fluoxetine; and c) a time interval of 10–28 h between the last fluoxetine intake and blood sampling. For patients with multiple TDM measurements of fluoxetine during the inclusion period only the most recent serum concentration analysis was included.

Patients were excluded from the study if a) serum concentrations of fluoxetine or norfluoxetine were non-detectable or outside the analytical quantification limits; b) *CYP2D6* genotype–phenotype translation was uncertain, i.e., gene duplication combined with the presence of a no function or a decreased function allele; c) the TDM request form mentioned use of any CYP-interacting drugs according to a compilation from Hiemke et al*.* [27]; and d) information from TDM request forms indicated conditions that could affect fluoxetine pharmacokinetics, i.e., pregnancy, gastric bypass surgery, or non-steady-state conditions – defined as less than 5 weeks since initiation of fluoxetine treatment.

Furthermore, to identify cases of treatment switch from fluoxetine to an alternative antidepressant, defined as a TDM measurement of another antidepressant drug replacing fluoxetine within one year after the last fluoxetine measurement, each patient’s TDM profile was reviewed longitudinally in the TDM database. Treatment switch was only studied in adult patients, since the number of switch events turned out to be limited in young patients, probably reflecting that fluoxetine is the preferred SSRI in children/adolescents.

### Fluoxetine and norfluoxetine analysis

The serum concentration analyses of fluoxetine and norfluoxetine were based on ultra high-performance liquid chromatography (UHPLC) and high-resolution mass-spectrometry (HRMS) developed for routine TDM analyses at the Center for Psychopharmacology. Samples were prepared by protein precipitation using a mixture of acetonitrile and methanol (9:1) containing the internal standards fluoxetine-d5 and norfluoxetine-d5. Purified samples were injected into a Vanquish UHPLC (Thermo Fisher Scientific, Waltham, MA). Chromatographic separation was achieved using an XBridge BEH C18 column (2.5 µm, 2.1 mm × 75 mm; Waters Corporation, Milford, MA) with a column temperature of 35º C. The mobile phase consisted of an ammonium acetate buffer (pH 4.8) and acetonitrile (20–95%). The run time was 3.50 min with retention times of 2.23 min for fluoxetine and 2.15 min for norfluoxetine. Detection was achieved with a Q Exactive Orbitrap HRMS (Thermo Fisher Scientific). The m/z values were 310.14133 for fluoxetine and 296.12568 for norfluoxetine. The range between lower and upper limits of quantification were 50–3000 nmol/L for both fluoxetine and norfluoxetine. Intra- and inter-day validation parameters of imprecision and inaccuracy were < 15%.

### CYP2D6 genotyping and phenotype subgrouping

Genotyping of *CYP2D6* was performed using TaqMan-based real-time polymerase chain reaction assays used for routine pharmacogenetic analyses at Center for Psychopharmacology. The assay included the no function alleles CYP2D6*3 (rs35742686), CYP2D6*4 (rs3892097), CYP2D6*5 (gene deletion), CYP2D6*6 (rs5030655), and the decreased-function alleles CYP2D6*9 (rs5030656), CYP2D6*10 (rs1065852), CYP2D6*17 (rs28371706) and CYP2D6*41 (rs28371725). Analysis of *CYP2D6* copy number was performed using TaqMan copy number assay targeting exon 9 with RNase P as endogenous control.

*CYP2D6* genotype/phenotype translation was performed according to guidelines from the Clinical Pharmacogenetics Implementation Consortium [28]. Homozygous carriers of no function variant alleles were defined as CYP2D6 PMs. Patients with a no function allele combined with either *1 or a decreased function allele were defined as CYP2D6 intermediate metabolizers (IMs), as were patients with two decreased function alleles. Homozygous carriers of *1 combined with allele duplication were defined as UMs. The remaining patients were defined as NMs, i.e., patients genotyped as homozygous carriers of *1 or carriers of *1 combined with a decreased function allele. Patients carrying a no function or decreased function variant allele in combination with allele duplication were excluded since the analytical method could not identify which allele was duplicated.

### Outcome measures and statistical analyses

Pharmacokinetic outcome measures were absolute serum concentrations of fluoxetine, norfluoxetine and active moiety (fluoxetine + norfluoxetine); dose-adjusted serum concentrations (C/D ratio) of fluoxetine, norfluoxetine and active moiety; and metabolic ratio (norfluoxetine/fluoxetine). Therapeutic failure was defined as a switch from fluoxetine to an alternative antidepressant, i.e. another drug within the SSRI group, a serotonin-norepinephrine reuptake inhibitor (SNRI), a tricyclic antidepressant (TCA), mirtazapine, mianserin, vortioxetine or bupropion, as verified by UHPLC-HRMS. A switch event was defined when a TDM analysis of a new antidepressant drug was performed within one year after the last included TDM analysis of fluoxetine. This was presumed to indicate either insufficient clinical response or side effects from fluoxetine treatment. Consequently, an antidepressant switch was used as a proxy marker for therapeutic failure, regardless of the cause.

IBM SPSS Statistics version 28.0.1.1 (SPSS Inc, Chicago, IL) was used for statistical analysis and graphical presentations. Continuous variables were reported as median (range). The associations between CYP2D6 phenotype and pharmacokinetic measures such as absolute concentrations, C/D ratios and metabolic ratios were examined using Kruskal–Wallis test followed by Bonferroni-corrected Dunn’s test using CYP2D6 NMs as reference group. To assess therapeutic failure in relation to CYP2D6 phenotype, the odds ratios (ORs) with 95% confidence intervals (CIs) for switching from fluoxetine to an alternative antidepressant was estimated between different CYP2D6 phenotype subgroups using CYP2D6 NMs as reference (chi-square test). A *p* value below 0.05 was considered statistically significant.

### Ethical considerations

The study was approved by the Regional Committee of the South-Eastern Health Authority in Norway (#482562) and the Diakonhjemmet Hospital Investigational Review board. Ethical approval was given without the requirement of patient consent since the study was based on existing data retrospectively retrieved from a routine TDM/pharmacogenetics service.

## Results

### Patient inclusion and characteristics

A total of 1430 patients met the inclusion criteria of the study. Thirty-two patients had non-detectable fluoxetine/norfluoxetine levels, 60 patients had fluoxetine/norfluoxetine levels outside quantification limits, 20 patients had inconclusive *CYP2D6* genotype, 78 patients used interacting drugs, 2 patients were pregnant, 2 patients had had gastric bypass surgery, and for 13 patients steady state serum concentration had not been achieved. Among the 78 patients excluded due to use of interacting drugs, the interacting drugs were bupropion (*n* = 43), valproate (*n* = 20), esomeprazole (*n* = 5), duloxetine (*n* = 3), omeprazole (*n* = 2), paroxetine (*n* = 2), levomepromazine (*n* = 1), phenobarbital (*n* = 1) and carbamazepine (*n* = 1). In total, 207 patients were excluded, leaving 1223 patients to be included in the study. Of these, 1027 were adult patients (age ≥ 18 years) and 196 were children/adolescents (< 18 years).

Population characteristics of the included adult patients are presented in Table 1. Among the adult patients there were 68 CYP2D6 PMs (7%), 347 IMs (34%), 587 NMs (57%) and 25 UMs (2%). The median age in the adult population was 36 yrs (range 18; 95), and 722 of the patients were women (70%). Among the 196 included children/adolescents there were 11 CYP2D6 PMs (6%), 80 IMs (41%), 101 NMs (51%) and 4 UMs (2%). The median age in the population was 16 yrs (9; 17), while 146 of the patients were girls (74%). There were no differences in patient characteristics between CYP2D6 phenotype subgroups for adults nor for young patients (Table 1, *p* ≥ 0.2).

**Table 1:** Patient characteristics of adult patients (≥ 18 years) and children/adolescents (< 18 years) according CYP2D6 phenotype subgroups

Adult patients (≥ 18 yrs)						
Characteristics	PMs (*n* = 68)	IMs (*n* = 347)	NMs (*n* = 587)	UMs (*n* = 25)	*p*	Total (*n* = 1027)
Women, n (%)	54 (79)	269 (78)	430 (73)	19 (76)	0.4	772 (75)
Age, yrs	35 (18; 95)	36 (18; 83)	36 (18; 94)	42 (18; 70)	0.6	36 (18; 95)
Fluoxetine dose, mg/day	40 (10; 80)	40 (10; 150)	40 (10; 150)	40 (10; 80)	0.4	40 (10; 150)
Sampling time, h	24 (11; 28)	24 (10; 28)	23 (10; 28)	24 (112; 27)	0.8	24 (10; 28)
Children/adolescents (< 18 yrs)						
Characteristics	PMs (*n* = 11)	IMs (*n* = 80)	NMs (*n* = 101)	UMs (*n* = 4)	*p*	Total (*n* = 196)
Girls, n (%)	6 (55)	59 (74)	79 (78)	2 (50)	0.2	146 (75)
Age, yrs	16 (11; 17)	16 (9; 17)	16 (10;17)	16 (14; 17)	0.9	16 (9; 17)
Fluoxetine dose, mg/day	20 (10; 40)	20 (5; 80)	20 (10; 60)	20 (10; 60)	0.3	20 (5; 80)
Sampling time, h	15 (10; 27)	16 (10; 28)	18 (10; 28)	23 (15; 25)	0.3	17 (10; 28)

*PMs* poor metabolizers, *IMs* intermediate metabolizers, *NMs* normal metabolizers, *UMs* ultrarapid metabolizers

Values are presented as median (range). P values are derived from chi-square and Kruskal–Wallis tests

### Pharmacokinetic measures

**Fig. 1:**
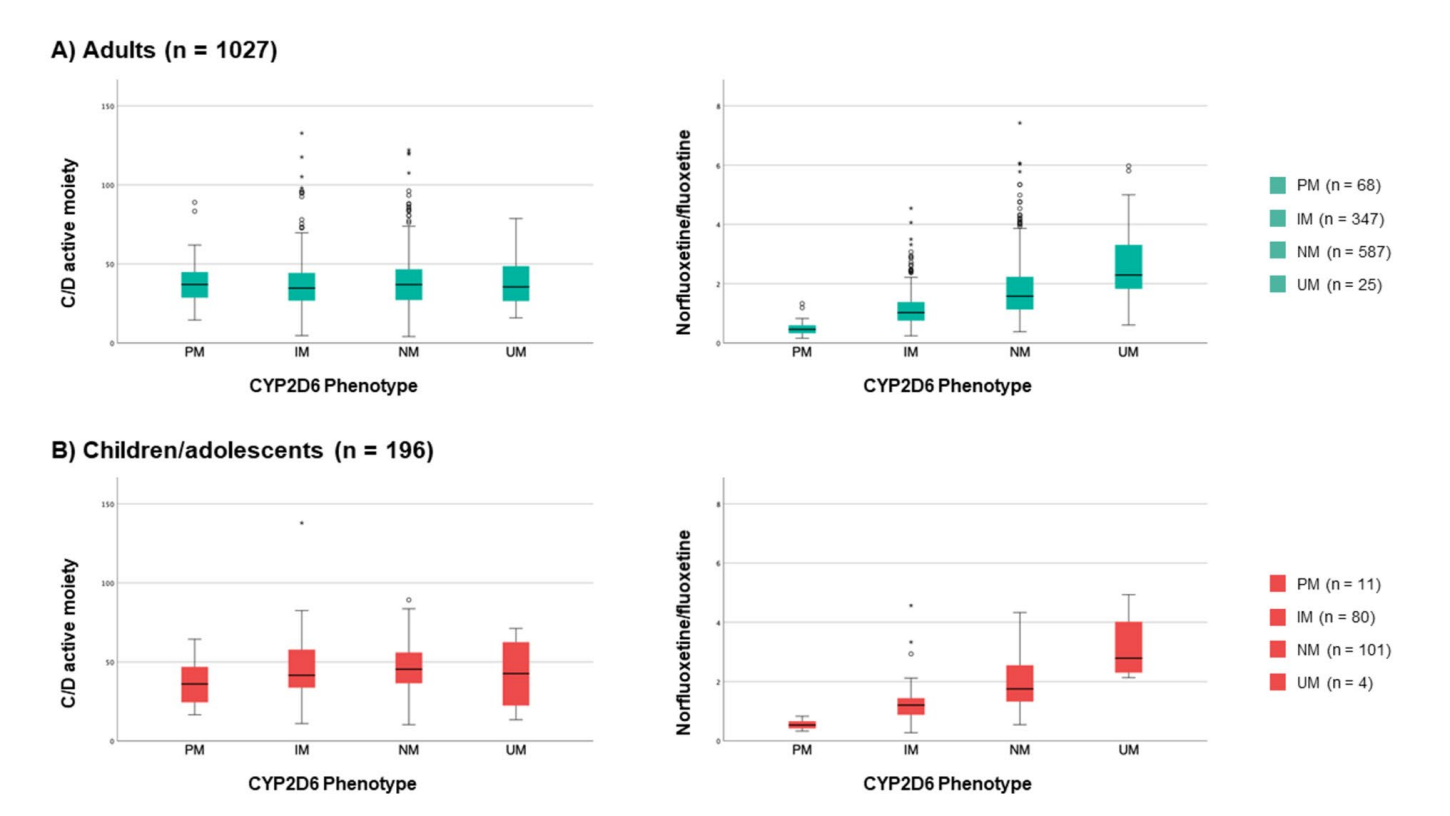
Boxplot of dose adjusted serum concentration of fluoxetine and norfluoxetine (C/D active moiety, (nmol/L)/(mg/day)) and metabolic ratio (norfluoxetine/fluoxetine) according to the CYP2D6 phenotype groups of poor metabolizers (PM), intermediate metabolizers (IM), normal metabolizers (NM) and ultrarapid metabolizers (UM) in **A**) patients ≥ 18 years (adults) and **B**) < 18 years (children/adolescents)

The pharmacokinetic measures of fluoxetine and norfluoxetine according to CYP2D6 phenotype subgroups are presented in Table 2. For adults, the C/D ratio of fluoxetine was 70% higher in PMs and 25% higher in IMs compared to NMs (*p* < 0.001), while the C/D ratios of norfluoxetine were 51% lower in PMs and 21% lower in IMs compared to NMs (*p* < 0.001). Similar trends were observed for the absolute concentrations of fluoxetine and norfluoxetine in adult patients (*p* ≤ 0.002). However, there were no significant associations between CYP2D6 phenotype and absolute concentrations or C/D ratios of the active moiety (*p* ≥ 0.1, Fig. 1). For the metabolic ratio of norfluoxetine/fluoxetine, CYP2D6 PMs and IMs had 69% and 38% (*p* < 0.001) lower metabolic ratios than NMs, respectively, while CYP2D6 UMs had 44% higher metabolic ratio than NMs (*p* = 0.04).

**Table 2:** Pharmacokinetic measures of fluoxetine and switch frequencies from fluoxetine to alternative antidepressants according to CYP2D6 phenotype groups

Adult patients (≥ 18 yrs)								
Pharmacokinetic measure	PMs (*n* = 68)	*p*	IMs (*n* = 347)	*p*	NMs (*n* = 587)	UMs (*n* = 25)	*p*	Total (*n* = 1027)
Fluoxetine concentration, nmol/L	809 (187; 2062)	< 0.001	512 (50; 2613)	0.002	434 (54; 2164)	358 (53; 1188)	0.6	482 (50; 2613)
Fluoxetine C/D ratio, (nmol/L)/(mg/day)	23.5 (8.0; 57.8)	< 0.001	17.2 (1.3; 70.7)	< 0.001	13.8 (1.8; 54.1)	11.6 (2.7; 23.8)	0.3	15.4 (1.3; 70.7)
Norfluoxetine concentration, nmol/L	339 (70; 1041)	< 0.001	538 (50; 1838)	< 0.001	681 (112; 2530)	856 (265; 2330)	0.1	597 (50; 2530)
Norfluoxetine C/D ratio, (nmol/L)/(mg/day)	10.5 (3.5; 35.6)	< 0.001	16.8 (2.5; 68.5)	< 0.001	21.3 (1.9; 84.2)	23.6 (10.3; 67.1)	0.2	19.1 (1.9; 84.2)
Active moiety concentration, nmol/L	1162 (257; 2995)	0.8	1042 (122; 3704)	0.2	1130 (166; 4459)	1413 (318; 3458)	0.9	1115 (122; 4459)
Active moiety C/D ratio, (nmol/L)/(mg/day)	37.0 (14.5; 89.0)	0.9	34.7 (4.7; 132.7)	0.1	37.0 (4.1; 122.1)	35.5 (15.9; 78.7)	0.9	36.0 (4.1; 132.7)
Metabolic ratio	0.5 (0.2; 1.3)	< 0.001	1.0 (0.2; 4.5)	< 0.001	1.6 (0.4; 7.4)	2.3 (0.6; 6.0)	0.04	1.3 (0.2; 7.4)
Switch to alternative antidepressant								
Frequency, n (%)	13 (19.1)		30 (8.6)		49 (8.3)	6 (24.0)		98 (9.5)
Odds ratio (95% CI)	2.3 (1.3; 4.0)	0.004	1.0 (0.7; 1.6)	0.9	N/A	2.9 (1.4; 6.1)	0.007	N/A
Switch to alternative antidepressant where genotyping was not performed within the switch period								
Frequency, n (%)	7 (10.3)		22 (6.3)		39 (6.6)	3 (12.0)		71 (6.9)
Odds ratio (95% CI)	1.6 (0.7; 3.3)	0.3	1.0 (0.6; 1.6)	0.9	N/A	1.81 (0.6; 5.5)	0.3	N/A
Children/adolescents (< 18 yrs)								
Pharmacokinetic measure	PMs (*n* = 11)	p	IMs (*n* = 80)	p	NMs (*n* = 101)	UMs (*n* = 4)	p	Total (*n* = 196)
Fluoxetine concentration, nmol/L	436 (230; 974)	0.5	452 (75; 2097)	0.2	357 (58; 1386)	205 (120; 310)	0.3	378.5 (58; 2097)
Fluoxetine C/D ratio, (nmol/L)/(mg/day)	23.5 (11.5; 36.2)	0.2	19.0 (4.4; 55.4)	0.01	16.6 (2.9; 38.3)	9.8 (4.3; 15.5)	0.4	17.8 (2.9; 55.4)
Norfluoxetine concentration, nmol/L	269 (94; 541)	< 0.001	488 (127; 1666)	< 0.001	678 (148; 1495)	570 (475; 767)	0.5	581 (94; 1666)
Norfluoxetine C/D ratio, (nmol/L)/(mg/day)	12.6 (4.7; 28.3)	< 0.001	20.9 (6.4; 82.5)	0.004	28.9 (7.4; 64.5)	31.1 (9.1; 59.2)	1.0	24.9 (4.7; 82.5)
Active moiety concentration, nmol/L	644 (331; 1515)	0.06	905 (241; 3243)	0.4	1059 (206; 2787)	759 (628; 1077)	0.8	990 (206; 3243)
Active moiety C/D ratio, (nmol/L)/(mg/day)	36.1 (16.6; 64.4)	0.5	41.5 (11.0; 137.9)	0.4	45.4 (10.3; 89.2)	42.6 (13.4; 71.2)	0.7	43.1 (10.3; 137.9)
Metabolic ratio	0.5 (0.3; 0.8)	< 0.001	1.2 (0.3; 4.6)	< 0.001	1.8 (0.5; 4.3)	2.8 (2.1; 4.9)	0.5	1.4 (0.3; 4.9)

*PMs* poor metabolizers, *IMs* intermediate metabolizers, *NMs* normal metabolizers, *UMs* ultrarapid metabolizers, *CI* confidence interval

Pharmacokinetic measures are presented as median (range). P values are derived from Bonferroni-corrected Dunn’s tests and from chi-square tests using NMs as the reference group

Among children/adolescents, CYP2D6 IMs had 14% higher fluoxetine C/D ratios than NMs (*p* = 0.01), but there was no significant difference between PMs and NMs (*p* = 0.2). There were no differences in absolute concentrations of fluoxetine between phenotype groups (*p* ≥ 0.2). For norfluoxetine, the C/D ratios were 56% lower in CYP2D6 PMs and 28% lower in IMs compared to NMs (*p* ≤ 0.004), and similar trends were observed for the absolute concentration of norfluoxetine (Table 2). Regarding the active moiety (fluoxetine + norfluoxetine), there was no significant association between CYP2D6 phenotype and neither absolute concentration nor C/D ratio (*p* ≥ 0.06), consistent with findings in adults. CYP2D6 phenotype was significantly associated with the metabolic ratio in young patients (*p* < 0.001). The metabolic ratio was 72% lower in PMs and 33% lower in IMs compared with NMs (*p* < 0.001), while no significant difference was observed between UMs and NMs (*p* = 0.5).

### Treatment switch

**Fig. 2:**
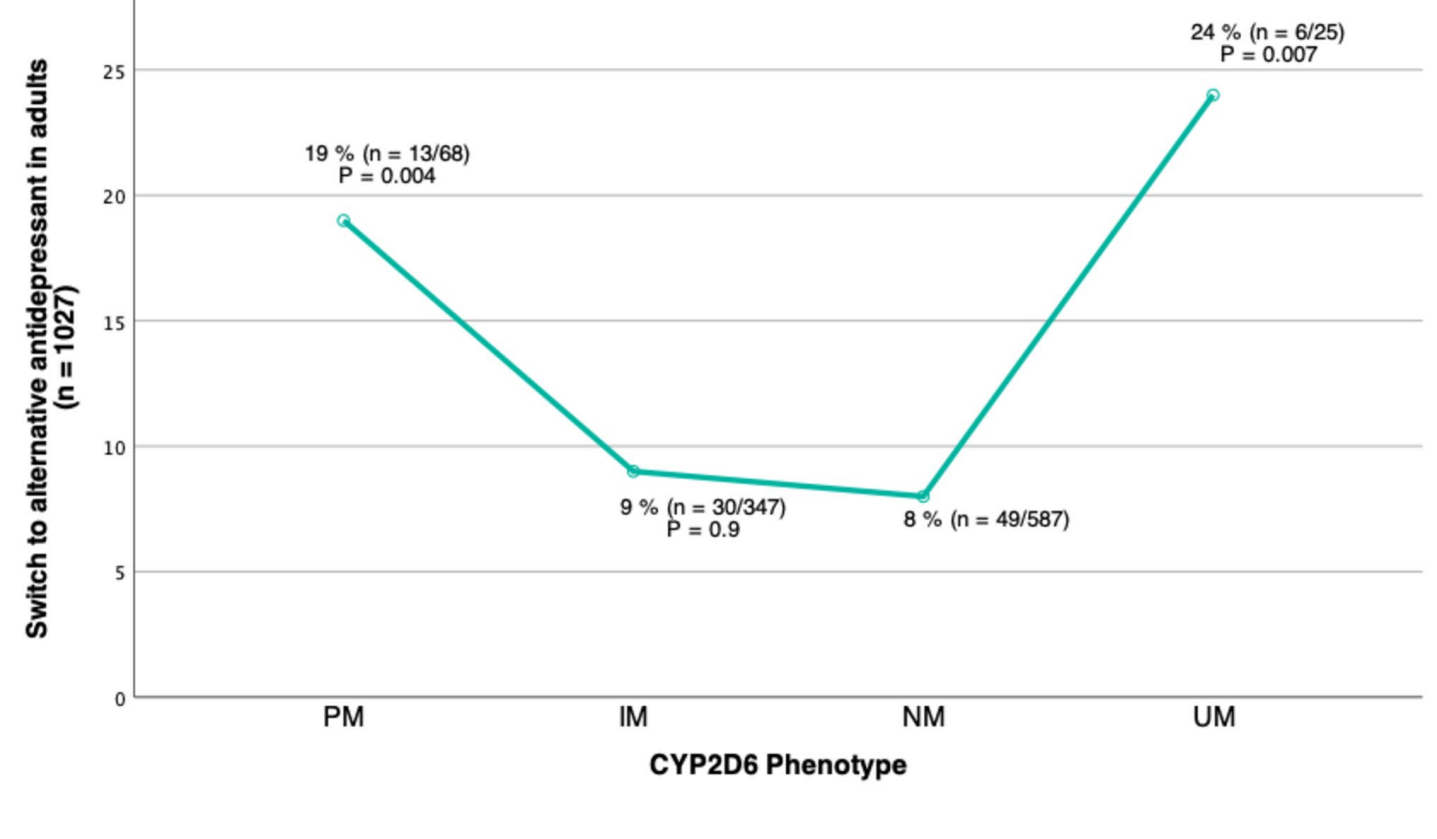
Percentage of patients who switched from fluoxetine to an alternative antidepressant according to the CYP2D6 phenotypes poor metabolizers (PM), intermediate metabolizers (IM), normal metabolizers (NM) and ultrarapid metabolizers (UM) in patients ≥ 18 years. A switch was measured as a serum concentration measurement of another antidepressant within 1 year after the last included serum concentration measurement of fluoxetine. P-values are derived from Chi-square tests comparing the odds for switching from fluoxetine to an alternative antidepressant for each CYP2D6 phenotype group to the NM group

A total of 98 switch events were identified among adults. The drugs replacing fluoxetine (number of events) were venlafaxine (*n* = 26), escitalopram (*n* = 18), sertraline (*n* = 13), bupropion (*n* = 13), duloxetine (*n* = 7), mirtazapine (*n* = 7), vortioxetine (*n* = 3), paroxetine (*n* = 2), citalopram (*n* = 2), clomipramine (*n* = 2), trimipramine (*n* = 2), fluvoxamine (*n* = 1), mianserin (*n* = 1), and nortriptyline (*n* = 1). As illustrated in Fig. 2, switch frequencies from fluoxetine to other antidepressants in adult patients were significantly higher for both CYP2D6 PMs and UMs compared to NMs. The odds ratios (ORs) for antidepressant switch were 2.3 for PMs and 2.9 for UMs compared to NMs (*p* ≤ 0.007, Table 2). There was no difference in switch rate between IMs and NMs (*p* = 0.9).

Pharmacokinetic measures were investigated in PM switchers vs non-switchers and in UM switchers vs. non-switchers (Supplementary Table). The median active moiety was 1262 nmol/L in PM switchers vs. 1154 nmol/L in PM non-switchers (*p* = 0.5), and 1144 nmol/L in UM switchers vs. 1459 nmol/L in UM non-switchers (*p* = 0.2). In the latter group of CYP2D6 UMs, a 50% higher norfluoxetine-to-fluoxetine metabolic ratio was observed in switchers than in non-switchers (*p* = 0.025). PM switchers and non-switchers had similar metabolic ratio (*p* = 0.9).

To evaluate whether treatment switch from fluoxetine to another antidepressant was dependent on the timing of *CYP2D6* genotyping in relation to the last TDM event of fluoxetine and the first TDM event of non-fluoxetine antidepressant, a sub-analysis was performed where patients genotyped between the last TDM measurement of fluoxetine and prior to the first TDM measurement of a new antidepressant were excluded. Among the 98 switch events previously identified, the minority of switch events (27 cases, 29%) had genotyping which occurred between the last fluoxetine TDM and first non-fluoxetine TDM, indicating that treatment switch was not driven by genotyping per se in the majority of the patients. A chi-square test of cases where genotyping had not been performed within the ‘switch period’, showed the same trends with higher switch frequencies in CYP2D6 PMs and UMs vs. NMs, but statistical significances were lost when reducing the population size (Table 2).

In children/adolescents, the number of switches per genotype group was too low for meaningful comparisons.

## Discussion

The present study shows that adult and young patients with decreased CYP2D6 metabolism have higher fluoxetine and lower norfluoxetine serum concentrations than patients with normal metabolism. The net result is unaltered sum of fluoxetine and norfluoxetine active moiety among CYP2D6 phenotype groups. These findings indicate that variability in CYP2D6 metabolism does not have an impact on dose requirements and clinical outcome of fluoxetine treatment. However, the fact that frequencies of fluoxetine switch to other antidepressants was significantly increased in CYP2D6 PMs and UMs may suggest that *CYP2D6* genotype is still of relevance for the clinical response of fluoxetine, although this speculation needs to be investigated in further prospective studies.

There is limited evidence regarding the association between *CYP2D6* genotype and fluoxetine pharmacokinetics. Some studies have reported that the metabolic ratio of norfluoxetine/fluoxetine is affected by CYP2D6 phenotype, which is supported by the present study [15, 22, 23]. In line with the present study, three previous studies – including less than 250 patients altogether – have reported no effect of CYP2D6 phenotype on the active moiety of fluoxetine and norfluoxetine [15, 22, 24]. The present real-world study included more than 1200 patients – whereof 79 were CYP2D6 PMs – and showed that PMs and IMs had higher fluoxetine levels and lower norfluoxetine levels than NMs. Based on the collective evidence regarding *CYP2D6* genotype and lack of effect on the exposure of fluoxetine active moiety, one would not expect that fluoxetine dosing requires individualization by *CYP2D6* genotyping.

As the total active moiety was unaffected by *CYP2D6* genotype, it is reasonable to speculate that the metabolic ratio between fluoxetine and norfluoxetine is of importance for overall effect and tolerability of fluoxetine treatment. The median norfluoxetine-to-fluoxetine metabolic ratio ranged from 0.5 in PMs to 2.3 in UMs (adults), which was paralleled by a two- to three-fold higher switch frequency compared with CYP2D6 NMs. While described as pharmacologically equipotent, fluoxetine and norfluoxetine still exhibit some differences in pharmacodynamic and pharmacokinetic properties with potential relevance for the clinical response. In vitro, it has been shown that fluoxetine exhibits a 1.8-fold higher serotonin-reuptake inhibitory (SRI) activity compared to norfluoxetine [29]. In addition, fluoxetine is more distributed to the brain than norfluoxetine, which may provide a stronger pharmacological response per serum concentration unit of fluoxetine vs. norfluoxetine [30]. Overall, one may therefore speculate that in CYP2D6 PMs, where the fluoxetine level was two-fold higher than norfluoxetine in the present study, the SRI activity may be substantially higher than in CYP2D6 NMs, that had a 1:1 relationship between fluoxetine and norfluoxetine. In CYP2D6 UMs, the fluoxetine level was only around 30% that of norfluoxetine.

Given the accuracy of the above estimates, the SRI activity will be expected to be significantly higher in PMs and lower in UMs vs. NMs, which ultimately may lead to increased SRI activity and side effects in PMs, while UMs may obtain lower SRI activity and reduced clinical response. However, these are speculations which need to be evaluated in prospective clinical trials. Furthermore, the complexity of potential differences in metabolic profiles and pharmacological contributions of fluoxetine and norfluoxetine enantiomers need further investigation to elucidate a potential difference in therapeutic efficacy and tolerability between CYP2D6 phenotype groups. Unfortunately, we were not able to distinguish between S- and R-norfluoxetine in the present study, as this requires enantioselective chromatographic separation which is not standard in TDM practice.

The autoinhibition mediated by fluoxetine and norfluoxetine on CYP2D6 metabolism complicates the assessment of CYP2D6 genotype effects on fluoxetine metabolism. This phenomenon will contribute to phenoconversion among patients with functional CYP2D6 metabolism, i.e. CYP2D6 NMs, IMs and UMs. However, a clear and highly significant difference in norfluoxetine-to-fluoxetine metabolic ratio consistent with genotype-predicted CYP2D6 activity suggests that CYP2D6 inhibition does not abolish an effect of genotype on fluoxetine metabolism. Thus, the present study’s aim of investigating the role of *CYP2D6* genotype on metabolism and potential treatment outcome of fluoxetine treatment had a valid fundament.

Only a few previous studies have examined the relationship between CYP2D6 phenotype and treatment response or side effects of fluoxetine [25, 26]. One of the studies reported poorer treatment effect and more side effects in 20 IMs compared to 81 NMs, although the only genetic variant which was included in the study was CYP2D6*4 [25]. The other study reported that ten patients with CYP2D6 PM phenotype did not have more adverse drug reactions from fluoxetine, nor did they discontinue treatment more often than 115 NMs [26]. Therefore, the evidence for an association between *CYP2D6* genotype and fluoxetine therapeutic outcome is scarce, and warrants further studies.

In the present study we were able to study adult and young patients separately. A previous study has investigated fluoxetine pharmacokinetic measures in 83 children/adolescents [23], but by only including one CYP2D6 PM patient its value is limited to evaluate the impact of CYP2D6 metabolism for fluoxetine pharmacokinetics in younger patients. Our study enabled inclusion of 11 PMs and 4 UMs of CYP2D6 providing robust statistical comparisons between the subgroups. While C/D ratios of fluoxetine did not differ significantly between PMs or UMs vs. NMs in younger patients, norfluoxetine C/D ratios were significantly different between PMs and IMs vs. NMs. There was no difference in the C/D ratio of active moiety across CYP2D6 phenotype groups. In summary, the C/D ratios of both fluoxetine, norfluoxetine and active moiety across CYP2D6 phenotype groups were similar in young and adult patients.

This study was based on real-world TDM data, inflicting important study limitations. The lack of access to medical records of the patients implied that switch events could not be assessed in relation to cause and treatment duration of fluoxetine. There is a risk that antidepressant switch was underreported with the current method, since TDM is not standard practice for antidepressants. However, TDM data are excellent in defining switch, i.e. if a drug is replaced by another one identified by serum analysis, it is certain that a switch event has occurred. Nonadherence to pharmacological treatment is an issue in real-world studies and may have influenced the assessment of fluoxetine pharmacokinetic variability. To avoid measurements likely to reflect poor adherence, TDM samples with drug levels below the lower limit of quantification were excluded. Other limitations comprise lack of information regarding factors such as comorbidity, organ function, body weight and concurrently used drugs not provided on the TDM request forms. These factors may have contributed to pharmacokinetic variability which the study was not able to account for. However, with the patient population of the study, including almost 80 CYP2D6 PMs in total, it is unlikely that the mentioned limitations would have changed the study outcome regarding effect of *CYP2D6* genotype on fluoxetine pharmacokinetic measures.

In conclusion, the present study shows that the active moiety of fluoxetine and norfluoxetine was unaffected by *CYP2D6* genotype, as patients without CYP2D6 had elevated fluoxetine and decreased norfluoxetine serum levels. Nevertheless, adult CYP2D6 PMs and UMs switched antidepressant treatment two to three times more often than NMs. This may indicate that the therapeutic outcome of fluoxetine is associated with relative levels of fluoxetine and norfluoxetine rather than active moiety in relation to CYP2D6 phenotype, but this warrants further studies.

## Supplementary Information

Below is the link to the electronic supplementary material.

Supplementary file1 (DOCX 16 KB)

## Data Availability

No datasets were generated or analysed during the current study.
