# Supplementary material for: Impact of CYP2D6 genotype on fluoxetine exposure and treatment switch in adults and children/adolescents
Source: Eur J Clin Pharmacol. 2025 Aug 8;81(11):1623–32. doi: 10.1007/s00228-025-03893-9 (PMC12511196; doi:10.1007/s00228-025-03893-9)
Supplement: Supplementary file 1 — Supplementary file1 (DOCX 16 KB) [file 228_2025_3893_MOESM1_ESM.docx]

**Supplementary Table** Pharmacokinetic measures of fluoxetine in switchers versus non-switchers stratified by CYP2D6 phenotype

| Adult patients (≥ 18 yrs) | | | | | | |
| --- | --- | --- | --- | --- | --- | --- |
| Pharmacokinetic measure | PM switchers  (n = 13) | PM non-switchers  (n = 55) | *p* | UM switchers  (n = 6) | UM non-switchers (n = 19) | *p* |
| Fluoxetine concentration, nmol/L | 979 (284; 1730) | 767 (187; 2062) | 0.6 | 254 (53; 418) | 354 (103; 1188) | 0.07 |
| Fluoxetine C/D ratio, (nmol/L)/(mg/day) | 26.6 (14.2; 53.4) | 23.4 (8.0; 57.8) | 1.0 | 11.0 (2.7; 17.9) | 11.9 (3.8; 23.8) | 0.5 |
| Norfluoxetine concentration, nmol/L | 422 (117; 751) | 336 (70; 1041) | 0.4 | 827 (265; 1342) | 908 (319; 2330) | 0.4 |
| Norfluoxetine C/D ratio, (nmol/L)/(mg/day) | 12.1 (4.7; 35.6) | 10.3 (3.5; 25.8) | 0.7 | 33.9 (13.3; 67.1) | 22.7 (10.3; 65.1) | 0.2 |
| Active moiety concentration, nmol/L | 1262 (409; 2377) | 1154 (257; 2995) | 0.5 | 1144 (318; 1573) | 1459 (422; 3458) | 0.2 |
| Active moiety C/D ratio, (nmol/L)/(mg/day) | 36.2 (20.5; 89.0) | 37.4 (14.5; 83.4) | 0.9 | 46.1 (15.9; 78.7) | 31.8 (20.8; 78.7) | 0.4 |
| Metabolic ratio | 0.5 (0.3; 1.2) | 0.5 (0.2; 1.3) | 0.9 | 3.1 (2.4; 5.8) | 2.1 (0.6; 6.0) | 0.025 |

PMs poor metabolizers, UMs ultrarapid metabolizers, C/D concentration-to-dose

Pharmacokinetic measures are presented as median (range). P values are derived from Mann Whitney U tests.
